# Older adults experience of transition to the community from the emergency department: a qualitative evidence synthesis

**DOI:** 10.1186/s12877-024-04751-6

**Published:** 2024-03-06

**Authors:** Brian Condon, Anne Griffin, Christine Fitzgerald, Elaine Shanahan, Liam Glynn, Margaret O’Connor, Christina Hayes, Molly Manning, Rose Galvin, Aoife Leahy, Katie Robinson

**Affiliations:** 1 School of Allied Health, Faculty of Education and Health Sciences, Ageing Research Centre, Health Research Institute, University of Limerick, Limerick, Ireland; 2 Department of Ageing and Therapeutics, University Hospital Limerick, Dooradoyle, Limerick, Ireland; 3 School of Medicine, Faculty of Education and Health Sciences, University of Limerick, Limerick, Ireland; 4 HRB, Primary Care Clinical Trials Network, Limerick, Ireland

**Keywords:** Older adult, Emergency department, Care transition, Qualitative evidence synthesis, meta-ethnography

## Abstract

**Aim:**

Older adults comprise a growing proportion of Emergency Department (ED) attendees and are vulnerable to adverse outcomes following an ED visit including ED reattendance within 30 days. Interventions to reduce older adults’ risk of adverse outcomes following an ED attendance are proliferating and often focus on improving the transition from the ED to the community. To optimise the effectiveness of interventions it is important to determine how older adults experience the transition from the ED to the community. This study aims to systematically review and synthesise qualitative studies reporting older adults’ experiences of transition to the community from the ED.

**Methods:**

Six databases (Academic Search Complete, CINAHL, MEDLINE, PsycARTICLES, PsycINFO, and Social Science Full Text) were searched in March 2022 and 2023. A seven-step approach to meta-ethnography, as described by Noblit and Hare, was used to synthesise findings across included studies. The methodological quality of the included studies was appraised using the 10-item Critical Appraisal Skills Programme (CASP) checklist for qualitative research. A study protocol was registered on PROSPERO (Registration: CRD42022287990).

**Findings:**

Ten studies were included, and synthesis led to the development of five themes. Unresolved symptoms reported by older adults on discharge impact their ability to manage at home (theme 1). Limited community services and unresolved symptoms drive early ED reattendance for some older adults (theme 2). Although older adults value practical support and assistance transporting home from the ED this is infrequently provided (theme 3). Accessible health information and interactions are important for understanding and self-managing health conditions on discharge from the ED (theme 4). Fragmented Care between ED and community is common, stressful and impacts on older adult’s ability to manage health conditions (theme 5). A line of argument synthesis integrated these themes into one overarching concept; after an ED visit older adults often struggle to manage changed, complex, health and care needs at home, in the absence of comprehensive support and guidance.

**Discussion/ conclusion:**

Key areas for consideration in future service and intervention development are identified in this study; ED healthcare providers should adapt their communication to the needs of older adults, provide accessible information and explicitly address expectations about symptom resolution during discharge planning. Concurrently, community health services need to be responsive to older adults’ changed health and care needs after an ED visit to achieve care integration. Those developing transitional care interventions should consider older adults needs for integration of care, symptom management, clear communication and information from providers and desire to return to daily life.

**Supplementary Information:**

The online version contains supplementary material available at 10.1186/s12877-024-04751-6.

## Introduction

An ageing population is presenting challenges for healthcare systems worldwide that are predicted to increase in the coming decades [1]. One such challenge is increasing demand for ED care [2]. Older adults account for 25% of all ED attendances and present with unique and complex needs and distinct patterns of service use compared to other population groups [3–5]. Upon discharge from the ED, older adults encounter high rates of adverse outcomes, including deconditioning, loss of functional independence, and ED readmission [6–8].

There is a growing focus on developing and evaluating interventions for older adults, in the ED, aimed at improving outcomes for this population such as case management, discharge planning, medication management/ safety interventions and geriatric ED’s [9]. However, a recent umbrella review revealed that the existing evidence regarding the effectiveness of these interventions in reducing adverse outcomes for older adults following ED discharge is limited. Consequently, the review recommended the urgent need for additional high-quality intervention studies in this area [10].

Further research on interventions for older adults that bridge the ED-community transition has been identified as a priority [9, 10]. Transitions of care are defined as “the various points where a patient moves to, or returns from, a particular physical location or makes contact with a healthcare professional for the purposes of receiving health care” [11]. This includes transitions between home, hospital, residential care settings and consultations with different healthcare providers in out-patient facilities” [11]. Transitional care encompasses a broad range of services including comprehensive assessment, patient and family education, staff handover and communication, medication reconciliation, and development of a care management plan [12, 13]. Two types of ED-based transitional care interventions were identified in a recent systematic review [14]. The first intervention type (individual needs assessment) comprised a structured comprehensive assessment of patient’s individual needs and care and addressing these through site specific or outpatient resources. The second type of intervention (individual discharge plan) comprised the coordination of care through an individualised discharge plan that summarises medication, treatment, and referral plan with patients, arranging transportation, referral to community services and initiating primary care referrals. Both interventions were associated with a significant decrease in hospital readmissions and ED revisits [14]. However, no study evaluated a combination of both intervention types and the authors note the need for further robust evidence of the effects of these interventions and evaluation of these interventions delivered in combination [14].

Given the limited available evidence on effective interventions to address the high rates of adverse outcomes older adults experience following an ED visit further intervention research is needed. Specifically, transitional care interventions warrant further evaluation. The need for future ED interventions for older adults to incorporate feedback from patients has been identified [9]. Qualitative research can provide valuable insights into patient priorities and the acceptability of interventions during intervention development [15, 16]. We identified several disparate qualitative studies reporting older adults’ experiences of ED discharge and transition to the community and we identified no synthesis of these studies. Thus, this study aims to synthesise existing qualitative evidence on perspectives and experiences of older adults’ care transition following discharge to the community from the ED.

## Methods

### Study aim

The aim of this study is to provide a comprehensive synthesis of qualitative literature reporting the experiences and perspectives of older adults’ transition of care following direct discharge from the ED to the community.

### Study design

A Qualitative Evidence Synthesis using meta-ethnographic methods [17] was used to conceptualise the experience of older adults transitioning from an ED to community setting. A study protocol was registered on PROSPERO (Registration: CRD42022287990). This study is reported in line with the eMERGe meta-ethnography reporting guideline [18].

### Reflexivity

All stages of the search and analysis were conducted by the lead author (BC) under supervision of the wider research team. The lead author is an occupational therapist and registered PhD candidate. They have 12 years clinical experience with older adults in hospital, ED and community settings. The lead author was a novice qualitative researcher at the outset of the synthesis and completed postgraduate training in qualitative research and study days on meta-ethnography and qualitative evidence synthesis with international experts. Members of the wider research team have clinical experience as a general practitioner (LG), geriatrician (MOC, ES, AL), physiotherapist (RG, CH), dietitian (AG), speech and language therapist (MM), nurse (CF) and occupational therapist (KR, BC) with experience of working with older adults. Several members of the research team have experience of conducting primary qualitative research and qualitative evidence synthesis (AG, MM, CF, CH, RG, RG, LG, AL).

## Identifying papers for inclusion and exclusion

### Search selection

A systematic literature search of six databases (Academic Search Complete, CINAHL, MEDLINE, PsycARTICLES, PsycINFO, and Social Science Full Text) was completed in March 2022 and updated in March 2023 by BC through EBSCOHost. In consultation with a specialist librarian in health research methods, a search strategy was developed based on three key concepts: ‘Qualitative research’, ‘Emergency Department’ and ‘Older Adults’ using MeSH terms. A search of a seventh database, Google Scholar, was also conducted using the three concepts as search terms and the first 10 pages of results were reviewed. Reference lists of included studies were searched for additional articles. Databases were searched from year 2000 and results were limited to those published in English. The search string is provided as supplementary file 1.

## Inclusion and exclusion criteria

### Articles meeting all the following criteria were included

1. Qualitative design reporting use of recognised methods of both qualitative data collection and analysis or mixed-methods study design where qualitative data could be extracted separately.
2. Participants aged 65 years or older. Samples including adults and older adults were included if findings on those aged 65 years or above could be extracted separately.
3. Participants discharged from the ED directly to any community setting (including home, nursing home, respite care, rehabilitation settings and own home).
4. Study findings report older adult’s perspectives and experiences of care and/or transitions of care following discharge from the ED.

### Protocol deviation

In the protocol registered on PROSPERO the research question focused both on the experience of transition to the community from the ED and older adults’ perspectives on outcomes of care. The concepts related to transition home were identified by the team as very rich and informative and a decision was taken to present analysis on perspectives of outcomes of care in a separate forthcoming manuscript.

### Selection procedure

Database search results were imported to Endnote X9 software [19]. Duplicates were removed and results uploaded to Rayyan software for screening [20]. Title and abstracts were screened independently against the inclusion criteria by two reviewers (BC, KR) and disagreements were resolved through discussion and involvement of a third member of the team where necessary (RG). Subsequently two reviewers independently screened full text articles (BC and KR) for final decisions regarding inclusion with disagreements resolved through discussion and involvement of a third reviewer where necessary (RG). The reference lists of all included studies were then reviewed to identify potential additional studies for inclusion.

### Critical appraisal and data extraction

The methodological quality of the included studies was appraised using the 10-item Critical Appraisal Skills Programme (CASP) checklist for qualitative research [21]. Two authors (BC, KR) independently assessed the quality of each study against this checklist. Disagreements were managed through discussion or involvement of a third member of the research team (RG). No studies were excluded based on quality; the appraisal was used to highlight methodological limitations in their interpretation of the study findings.

Two authors (BC, KR) of the team extracted data from included studies independently. This was completed using a custom pre-piloted data extraction form. Data extracted included: author, year of publication, country, sample, data collection setting, sampling method, data collection, and major findings.

**Fig. 1:**
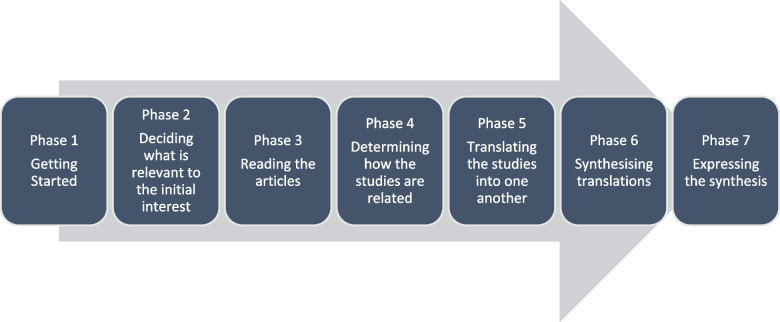
The seven phases of meta-ethnography

A seven-step meta-ethnographic approach was used to synthesise findings across all studies [17]. This approach was selected as it allows synthesis of findings from multiple studies and development of a higher order interpretation [22, 23]. The first phase ‘getting started’, involved the development of the research question and setting of inclusion criteria with the wider research team. The second phase focused on ‘deciding what is relevant to the initial interest’. A consensus on the inclusion criteria was reached between the research team and searching for the research articles was completed followed by quality appraisal of each included article. In the third phase, ‘reading the articles’, all included articles were read and re-read by two reviewers and extracted second order constructs were imported into NVivo Version 12 Pro software [24]. In phase four, ‘determining how the studies are related’, a grid of key concepts (second order concepts) was developed using the extracted data. The key concepts from each study (phrases or themes) were then juxtaposed against one another by two members of the research team allowing common and recurring concepts to be identified. In phase five, ‘translating the studies into one another’, two researchers took the identified concepts and compared each concept with all other concepts to identify similarities and differences across concepts and to enable organisation of the concepts into further conceptual categories (presented in the findings section as themes). This process was similar to the process of constant comparison and allowed the research team to stay close to the meaning and contexts of the original studies. Throughout the analysis process the important characteristics of included studies were reviewed to contextualise findings and to inform research team discussions. Across the included studies second order concepts were reciprocally translatable. In phase six of analysis, ‘synthesising translations’, a line-of-argument was synthesised from the conceptual categories identified in phase five. Finally, phase seven ‘expressing the synthesis’ follows below according to the eMEGEe meta-ethnography reporting guideline which is provided in supplementary file 2 [18] and PRISMA flow diagram [25]. Figure 1 present the seven phases of meta-ethnography. Ethical approval was not required for this synthesis.

### Public and patient involvement

The concept of this study was informed by an established Public and Patient Involvement (PPI) stakeholder panel of older adults and family caregivers [26]. This PPI panel has had experience from several ED related studies over a three-year period. The PPI panel were also involved in interpretation of findings. When data analysis was complete, and preliminary third order constructs were developed, a 2-hour long meeting was scheduled with seven older adults / family caregiver members of the PPI panel. The meeting was facilitated by four members of the research team (BC, KR, RG, CH) and involved a presentation in lay language of the research process and preliminary third order constructs. A facilitated discussion found unanimous agreement with the researchers’ interpretation and presentation of findings that was in keeping with older adults / family caregivers’ own experiences of transition home from the ED. A line of argument was collaboratively developed through discussion between the research team and PPI panel members.

### Findings

**Fig. 2:**
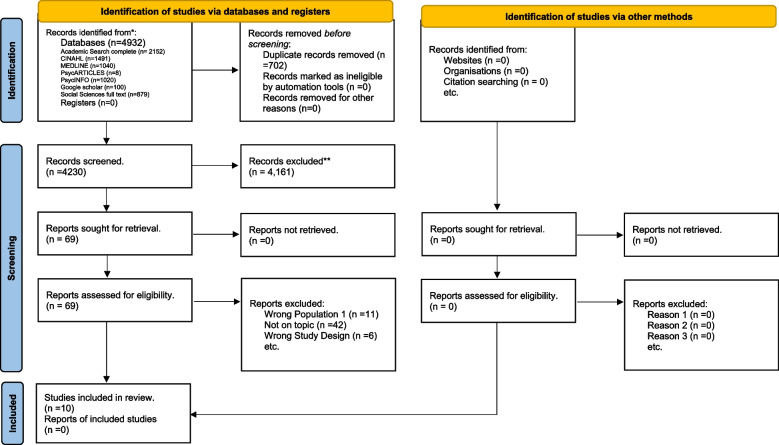
PRISMA flow diagram

The initial and updated searches in March 2022 and March 2023 yielded 4932 results; 702 duplicate articles were removed. Subsequently, 4230 articles were assessed for eligibility for inclusion. Of these, 69 articles were screened based on full text with 10 articles were included in the final review. This is outlined in the PRISMA flow diagram in Fig. 2.

### Characteristics of the included studies

The 10 studies included in this synthesis represented the experiences of 334 older adult participants. A table presenting the characteristics of the included studies is provided in supplementary file 3. Included studies were published between 2014 and 2022. Nine of the 10 studies included more women than men [27–35].

All included studies reported on experiences of older adults’ discharged from the ED to diverse community settings including their own home/with other family members [27, 28, 33–36], retirement home [33], care home [35], senior living facility [36], assisted living [33] and sheltered accommodation [35]. Details of older adults’ discharge destinations reported in the included studies is provided in supplementary file 4. Of the 10 studies, five studies did not put any constraint on cognitively impaired older adults being included in the study [27–29, 31, 35]. Eight studies recruited older adults with any diagnosis, one study recruited only those who had two or more chronic diseases [27] and one recruited only those with frailty [35]. One study recruited those older adults who frequently attended the ED [32] and one recruited older adults who had experienced an intervention designed to improve discharge practices [34].

All studies were conducted in high income settings. Five of the included studies were conducted in Canada [28, 29, 31, 33, 36], two in Denmark [27, 34] and one each from the United Kingdom [35], the Netherlands [32] and United States of America [30]. Recruitment was conducted at 17 ED sites across the 10 studies. In terms of participant recruitment methods, nine studies recruited participants during their ED admission and one study used announcements and postings for participant recruitment [31]. Semi structured interviews were the most frequent data collection method used with only two studies using a survey or questionnaire [28, 33]. Of note, no study collected data through observation.

### Quality appraisal

Results of quality appraisal are presented in supplementary file 5. No studies were excluded based on quality as all included studies contributed important information addressing the research question. Only four studies reported the process of collecting data in a manner that addressed the research issue. In many cases insufficient description or justification for the timing or methods of data collection were presented.

### Themes

**Fig. 3:**
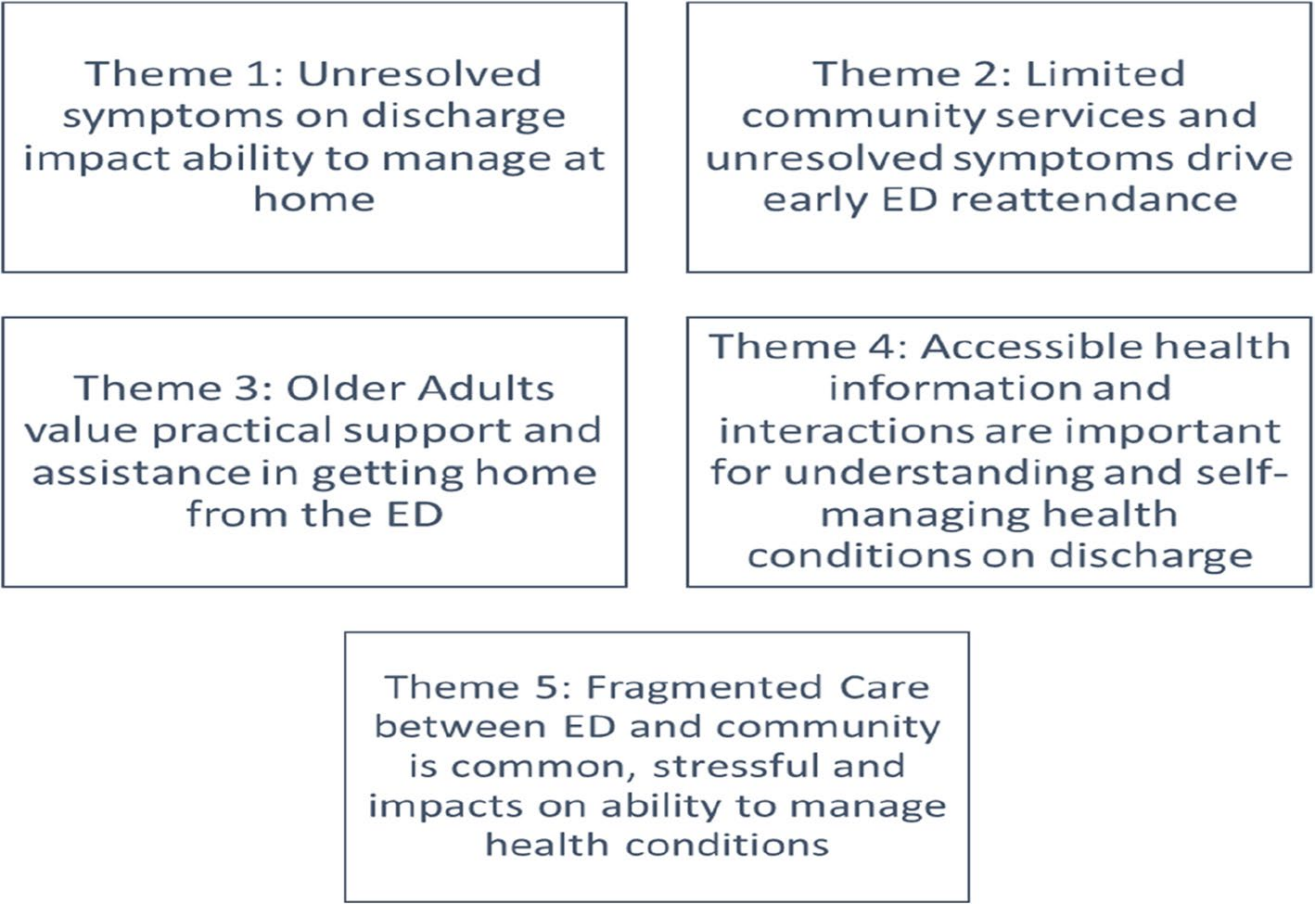
Themes developed through the meta-ethnographic process

Five themes were developed through the meta-ethnographic process (see Fig. 3 for details).

### Theme 1: unresolved symptoms on discharge impact ability to manage at home

This theme relates to older adults’ experiences of unresolved symptoms at the point of transition to the community from the ED. Although older adults wanted to feel well before discharge, reports of unresolved symptoms were common across the included studies and led to difficulties managing at home, such as difficulty moving around their own home [34], walking [36], completing domestic tasks such as cleaning, laundry [30, 32] and completing personal care tasks [30].

Eight studies reported on older adults’ experience of unresolved and undiagnosed symptoms at the point of transition to the community [28–30, 32–36]. Older adults often described their discharge from the ED as being rushed, unanticipated or occurring before their symptoms had resolved or before a diagnosis was received [28, 30, 32–36].

“If they could have kept me longer...because I was given my discharge on Monday and I am still suffering from the same problems” [28].

Symptoms experienced by older adults on discharge often varied. However, pain [28, 34–36] and mental health challenges were reported in several studies [28, 29, 32, 34, 36]. These symptoms often had a negative psychological impact on older adults and led to reported feelings of confusion [32], insecurity [32], self-doubt [32], uncertainty [28], worry/anxiety [28, 29, 32], disappointment [27, 29, 32], feeling down [29] and frustration as to why their symptoms had failed to resolve when transitioning to the community [32, 34].

“It’s a bit weird and crazy, but I do not dare to go outside! I am too afraid, but I do not understand why. I panic at the thought of it. However, if I went upstairs, then I did not remember anymore why I was going there. It was probably my own insecurity, I was confused, and I just did not remember things well. That was really disturbing and worrying” [32].

“I was really very down temporarily about this. Thinking, oh, you know, this is never going to get better” [29].

Older adults reported that unresolved symptoms impacted their overall ability to manage at home [28, 29, 32, 34–36]. Unresolved symptoms also led to reports of discomfort [29, 34], limited mobility [34–36], fatigue [28, 32, 34] and difficulty sleeping [36].

Older adults reported having difficulty returning to their functional baseline and managing everyday tasks when discharged home from the ED primarily due to unresolved symptoms [27–30, 32–34]. They reported needing to make significant changes to their way of living/lifestyle [27, 29, 34] and needing increased home supports from family/caregivers or, in certain circumstances, a period of rehabilitation [27, 28, 33, 34]. Additionally, older adults described the need to adapt their way of living by resting during the day [30, 34], using adaptive devices or equipment [27, 29, 34], discontinuing their usual hobbies [34], and increased reliance of family/caregivers [28, 30, 33]. Older adults in one study discussed the impact of their ED attendance and illness on their informal caregivers’ jobs, finances, family lives and social lives [30]. Fear of symptom recurrence also led to restrictions in daily activities that might trigger symptoms [30].

“I cannot do anything. No, I can walk a bit. I sit a lot. Vacuuming, cleaning, that sort of things, I cannot do that. Yes of course, I am not happy about it. I do not see any progression in my recovery and I expected that. Because, until now...so far things are not so positive” [32].

“They give me equipment so I could get around and maintain my lifestyle and gradually get back to where I was” [29].

### Theme 2: limited community services and unresolved symptoms drive early ED re-attendance

This theme related to older adult’s experience of ED reattendance within a short period of time, often within a small number of weeks, after transition from the ED to the community. Reasons for older adults re-attending the ED varied however, the primary reasons included: continuation or re-occurrence of symptoms that caused initial ED attendance, inadequate healthcare alternatives and limited community services.

Six of the 10 included studies reported experiences of older adults’ readmission to the ED shortly after being discharged to the community [28, 31–33, 35, 36]. In one study older adults noted that they had greater trust in the care they received in the ED than the care they received from their own GP [32].

“So I had to come back about 5 hours later…for the very same problem” [36].

Un-resolved symptoms at the point of ED discharge, re-occurrence of symptoms shortly after discharge or lack of understanding about their symptoms were identified by older adults as primary catalysts for ED reattendance [28, 32, 33, 35, 36]. In certain instances, re-attending the ED had become part of the older adults’ care routine however, they reported that they were happy to reattend the ED again [36].

“I left for home with no answer, I don’t know why the pain, I don’t know anything. So, I came back the second time, to get answers for the first time” [36].

Within included studies, older adult ED reattendance was not related to their care received in the ED but, catalysed by limited community services or care alternatives available [28, 32, 35, 36]. These included limited home care support received on discharge from the ED [35], delays in obtaining a general practitioner (GP) appointment [36], or being advised that their health condition was not manageable by a GP [36]. These experiences of frequent ED attendances had an impact on both the older adults and their partner’s quality of life [32].

“We gave up Nordic walking even though we love it. With all those ED visits, we are exhausted and it is too difficult for my husband. We have been so many times to the hospital for ED and outpatient visits. We are just so burnt out from it all” [32].

Older adults describe being advised to reattend the ED by their GP [36], family members/caregivers [32], specialist care services [32, 36] and residential home care staff [36]. Older adults reported that they received advice to re-attend the ED for various reasons; including severity of their symptoms [36], lack of receipt of a discharge letter from the ED [32], or their condition being beyond GP competency to manage [36]. Additionally, in certain circumstances older adults described conflicting advice being received from health care providers as to whether their condition required an ED attendance or not [33].

“I was discharged home and instructed to come back if I had a fever. So, we went to the ED when I had a fever, but they could not find anything. So I was sent home and the fever returned. I went back to the ED several times as the doctor told me to” [32].

### Theme 3: older adults value practical support and assistance in getting home from the ED

This theme relates to older adult’s desires for safe and planned discharge with organised transport to the community. To ensure a positive and safe discharge home, older adults expressed a desire for a planned discharge and a desire for health care providers to communicate their needs and time of discharge to transport providers and ensure necessary assistance is available on ED discharge.

Older adults value being informed of when discharge is due to happen [35]. They reported that they prefer to be discharged home during daytime hours and when a caregiver or family member is present [35]. However, older adults reported not always being asked if they required assistance or had assistance when being discharged from the ED [28, 35]. Additionally, older adults describe the discharge process as very long, and distressing, with delays in receiving medication/discharge letters/porters/transport [35]. Older adults in one study describe being unhappy when no assistance with arranging transport home was provided [32].

“After being discharged from the emergency department, I found that I had to figure things out myself. The exit, I didn’t know where it was. I had to inform myself, talk to people, and find the exit, take a cab to get back home. The thing is when you get there, you are by yourself, you’re here for the first time. It isn’t obvious” [28].

Older adults described negative transport related experiences when transitioning to the community from the ED [27, 28, 35] including experiences of being discharged home late in the evening, being woken up early in the morning, being taken to the discharge lounge in their night clothes, uncomfortable waits for transport [35] and not receiving the required assistance to get into their homes, for example from taxi drivers [27, 35]. In one study, availability of appropriate transport was described as an important factor for older adults attending follow up appointments and senior-friendly parking was also highlighted as influencing the older adult’s ability to follow through with ED recommendations after transition to the community [33].

### Theme 4: accessible health information and interactions are important for understanding and self-managing health conditions on discharge from the ED

This theme relates to the often inadequate communication from healthcare providers experienced by older adults at the point of discharge and transition to the community from the ED.

In three studies, older adults report wanting to be actively involved and included in their medical care [27, 32, 35]. However, this desire was in some cases hampered by communication difficulties and challenges with healthcare providers. Older adults describe health care professionals not listening to them [31, 34] and failing to adjust their communication methods/style to compensate for older adults’ sensory deficits [31].

“I also can’t see very well, I’m told. I have macular and a hearing aid...[providers] have to make sure that they can hear and you have to make sure that they can see....They hand you something and leave, they’re gone. So you sit there with a paper in front of you and don’t know what to do” [31].

In some cases, older adults reported having difficulty asking necessary questions to health care professionals [33, 35]. Additionally, older adults in one study perceived health care professionals did not identify when they did not understand information during their ED admission [33]. Thus, older adults describe having further questions about care recommendations on ED discharge [29, 30, 33]. Older adults described the need for follow up questions to be answered following discharge to the community from the ED [29, 32] when they were less stressed [32] or to be provided with reassurance that they could access ED again as needed [29].

Older adults described in one study that healthcare professionals provided good information on further actions and examinations [34] however, this was not a consistent finding within this study. In six of the studies, health care professionals’ communication with older adults on discharge was described as insufficient by older adults [28, 30, 32–35]. This resulted in older adults leaving the ED in some cases with incomplete discharge instructions, limited information on their medical symptoms on discharge [30, 32], insufficient information on future medical plans after their transition to the community [28] and unanswered questions [35].

Within included studies the format of communication at discharge was also identified as an issue [32, 34]. Older adults describe being provided with only verbal advice or instructions on discharge [34] however, older adults reported that verbal instructions were not effective nor was it their preferred communication method [32–34]. Older adults report difficulties in recalling verbal information or advice once discharged to the community without written information being received [32–34], feeling overwhelmed by too much information [33] and having difficulty hearing verbal instructions [33].

“I thought I understood about how to take the eye drops, but I once I got home I couldn’t remember all the things they told me to do” [33].

“It would be nice to get something in writing. That’s always nice, so you can return to it. You can’t do that when it is oral. That’s way I say, you should be two instead of one. But if you are alone, you can in doubt about what it was. It is very different if you get it in writing—then you can go back” [34].

Written communication/instruction that include specific details related to care and devoid of medical jargon was identified in several studies as older adults’ preference in addition to any verbal information at ED discharge [31, 33]. Included studies did provide examples where older adults were provided with written instructions on discharge [28, 30, 31, 34]. However, criticisms of the written information provided included difficulty understanding the information [34], no explanation of the written information [28, 31], a lack of specific treatment information e.g., medication changes [34], provision of information that older adults did not require [28], or written information being cumbersome, lengthy and unhelpful [30].

“I haven’t had a chance to look at that paperwork. I have to look at it later. She told me about it and showed it to me, but it was just so much” [30].

Conflicting recommendations or information from health care professionals were reported by some older adults [28, 30, 33]. Older adults describe being provided with conflicting information related to their medication management [28] and whether their health concerns required an ED admission versus being managed in the community [33].

“They contradicted the 2 doctors...one who said ‘you have a cyst behind, but you have to stop the Coumadin and then we will operate you...And then the other said: no, no, no, we must not stop, we must not stop the Coumadin, it must continue. So, that’s it, they both contradicted each other” [28].

### Theme 5: fragmented care between ED and community is common, stressful and impacts on ability to manage health conditions

This theme relates to older adults’ experiences of fragmentation and discontinuity of care during their transition to the community. Care co-ordination between the ED and the community was experienced by some older adults. However, fragmented care was the more prevalent experience reported by older adults relating to medication management, outpatient appointments, follow up care and scheduling of outpatient diagnostics. Older adults reported that, fragmented care can lead to negative feelings of stress and uncertainty and difficulty managing health conditions.

In six of the included studies, there were mixed experiences of healthcare coordination reported by older adults. Follow-up appointments after discharge from the ED were arranged for some but not consistently for everyone in these studies [27, 28, 31–33, 35]. One study reported seven of the 15 participants experienced continuity of care based on interviews with older adults in the ED and a second interview 4–12 weeks after discharge [27]. In a further study older adults on specific discharge care pathways from the ED had experiences of well-planned follow up appointments and treatment of care [32]. Two studies highlighted where older adults had a positive experience where follow up calls occurred and useful information or resources were provided to them [31, 33] and follow up appointments were arranged quicky after ED discharge [33]. In a further study older adults suggest the need for follow up calls after an ED index visit would be helpful [30].

“I would say all, the reception, the staff, everything there was very very very well orchestrated. It’s excellent what I was given as advice and for follow-up especially. I learned to do everything” [28].

“I had a number of follow up calls from different people. They gave me excellent information, they provided me with all kinds of resources” [33].

However, other older adults within included studies describe experiences of fragmentation and discontinuity of care following discharge from the ED [27, 28, 30, 32, 34, 36]. Older adults describe wanting clear information on their discharge plans when transitioning from the ED to the community [35]. However, older adults report experiences of not being provided with sufficient information about further planned medical diagnostics, follow up appointments and the wider care process when transitioning from the ED to the community [28–30, 32, 34]. In one study, older adults described waiting for further diagnostics on discharge as a long and difficult process, leading to delays commencing necessary treatment [34]. A common issue reported was lack of clarity on who was organising follow up appointments [28, 30, 32].

“I don’t know if she [emergency clerk] will call me to set up the appointment or if the rheumatologist office will, I don’t know. I am missing some information, but I didn’t receive any paper or prescription” [28].

“We are disappointed. We did not realize that we were responsible for organizing the follow-up appointment with the outpatient clinic. We thought the ED would do that” [32].

“They called me after I’d come home and said, “There is a lump in your parotid gland, and you need to get that looked at right away.” Unfortunately, they did not give me any information. Do I contact an ENT? Do I contact a neurologist? I had to fumble around until I found the right doctor to see. That can be a problem, not giving people adequate follow-up, directing them where to go, I think” [30].

Older adults reported feeling stressed [29, 32], anxious [29], disappointed [32], unsafe [32], confused [28, 34], and uncertain [28] due to the fragmentation of care, delays, or insufficient information about their care on discharge. Older adults frequently had unscheduled reattendance to the ED before follow-up appointments occurred [36]. In some instances, when follow up care was delayed, older adults sought advice from helpline numbers for non-urgent issues or considered attending a private clinic [28].

Older adults reported experiences of fragmented medication prescription/management [28, 35, 36] and they described changes made to their medication regime by ED staff were insufficient [28] or no information was provided to their GP on these medication changes [35]. In one study, some older adults described leaving the ED without a medication prescription [36]. In some cases, medication changes were not executed by the GP due to fragmentation of care [35]. Older adults themselves reported also being unaware of the rationale for medication changes, side effects of their medication and medication instructions when being discharged to the community [28, 35, 36]. In two studies, community pharmacists were reported to provide information on medication side-effects and potential interactions [28, 36].

“I learned them [potential side effects] when I received the antibiotics with the letter including all information. Yes, at the pharmacy” [28].

“I got the prescription filled…but my pharmacist checked, and said it would have a very bad interaction with one of my blood pressure pills” [36].

### Line of argument

We identified one concept that unified the identified third order constructs; after an ED visit older adults often struggle to manage changed, complex, health and care needs at home, in the absence of comprehensive support and guidance. Some older adults reported positive, supportive experiences during this transition however, difficult experiences were most commonly reported across the included studies. Key areas for greater support and guidance are comprehensive support with transport home and support managing symptoms and daily activities once home, enhanced guidance and communication from healthcare providers and enhanced integration of care during their transition of care from the ED to the community.

## Discussion

This synthesis of qualitative studies exploring older adults’ experience of transition to the community from the ED found that older adults reported unresolved symptoms on discharge (theme 1) which negatively affected their return to daily life. Unresolved symptoms, along with limited community services, were a driver of early ED reattendance for some older adults (theme 2). Although older adults value practical support and assistance in getting home from the ED this was infrequently provided (theme 3). Inaccessible health information and inaccessible interactions with healthcare providers (theme 4) and fragmented care at the point of transition home (theme 5) were commonly reported by older adults.

In response to population ageing, policy makers have strongly endorsed a shift towards integrated care for older adults [37]. Integrated care is “an approach that promotes collaboration across organizational and professional boundaries to provide more connected care for patients, family and caregivers in their local communities” [38]. An integrated care approach promotes high quality transitions across different healthcare systems [38]. This synthesis of 10 studies revealed that fragmented care is the norm for older adults when transitioning to the community from the ED underscoring the need for care integration.

Care Integration has been defined as a hierarchical concept of three different levels of continuity across disciplinary and organisational boundaries: informational continuity (information ‘linking care from one provider to another and from one healthcare event to another’) [39], management continuity (services for a health condition are delivered in a responsive, ‘complementary and timely manner’) [39], and relational continuity (an ongoing therapeutic relationship between a patient and one or more providers) [39]. These are of particular importance for complex co-morbidity and chronic disease which are common in older adults. Fractures in all three levels of continuity were evident in the findings of our synthesis. In relation to informational continuity, fractures in this concept were exemplified by discontinuities around medication prescription and management, outpatient appointments and scheduling of further diagnostics. Older adults describe experiences of management discontinuities such as reports of reattendance at the emergency department before following up appointments or care occurred. Relational discontinuities included non-sharing of treatment/management information, lack of, or inappropriate information received. It is important to note that solutions to care integration do not lie exclusively in the ED and require involvement of responsive primary care and community partners to respond adequately to older adults changed health and care needs after an ED visit.

Polypharmacy is a growing challenge driven by the ageing demographic. A qualitative study of older adults and family carers experiences of post hospital discharge medicines management, reported a perception by older adults of disorganised and chaotic communication between the hospital, community pharmacy and primary care provider [40]. Medication related errors or omissions were common when older adults were transitioning from one care setting to another [41]. Our findings clearly support a need for interventions to support medication continuity at the point of ED discharge. In line with this and the policy shift towards integrated care, a systematic review found that interventions that best support older adults’ medication continuity are those that bridge transitions of care [41].

Fractured and delayed communication between ED and community healthcare providers were commonly reported across the included studies leading to negative consequences for older adults including treatment delays and stress. Health Information Exchange (HIE) involves the seamless electronic transfer of health information between healthcare organisations or different healthcare settings (for example between hospitals and community services) and HIE shows promise for improving care co-ordination and reducing both duplication and costs [42, 43]. To date HIE research has been dominated by the ED setting as a technology solution that allows healthcare providers rapidly access information from other organisations aligns with ED information needs [43], although HIE is relevant to all clinical settings. Despite the established benefits of this technological solution, HIE deployment across international settings including the five countries where studies included in this review were conducted is variable [44–46]. Notably, Canadian healthcare providers report low rates of electronic health information exchange with other providers [47]. This is important as four of the included studies in this synthesis were conducted in Canada. Many factors are needed to enable HIE including political support, financial resources, technologies and addressing implementation barriers [46] in complex and fragmented healthcare systems.

Unresolved symptoms, particularly pain and mental health challenges following discharge were commonly reported and had a negative impact on older adults’ function. Interviews with 46 patients seeking care in the ED about expectations of care identified the importance of symptom relief, having a plan to manage their symptoms, understanding of the cause and expected trajectory of their symptoms and reassurance [48]. Similarly, a qualitative study of people with diabetes mellitus or cardiovascular disease who were being discharged from an ED found that, their primary expectation about an ED visit was receiving a diagnosis and reassurance. Post-discharge participants greatest need was answers about the cause of their symptoms and knowledge about what to expect [49]. In our synthesis, unresolved symptoms were common, troublesome, affected return to everyday activities and older adults reported that unresolved symptoms are a major driver of early ED reattendance.

Reattendance at the ED has been identified as a frequent adverse outcome experienced by older adults following transitions of care [50]. We found that symptom reoccurrence, lack of diagnostics on initial ED visit and inability to access needed community services were key drivers in older adults’ requirement to reattend the ED. Interviews with 60 people who had an unscheduled return to the ED within 9 days of an index ED discharge found that, the primary reason for ED reattendance was fear and uncertainty about their health condition [51]. Additionally, a further study identified that over half of older adults did not understand discharge information about expected course of illness as well as return precautions and 42.3% of patients had returned to the ED within 90 days of discharge [52]. The findings of our synthesis suggest that ED healthcare providers should explicitly discuss expectations about symptom resolution during discharge planning and where possible support continuity of care in addressing ongoing symptoms post discharge to the community.

A questionnaire study conducted the day after discharge from a Finnish ED (*n* = 132) found that written instructions were only provided on discharge to 12% of participants (the majority were aged 50–79 years) [53]. This reflects our findings as older adults in the majority of studies did not report provision of written discharge instructions thus, they were unable to recall recommended ED interventions once discharged. Older adult’s safe transition of care is heavily influenced by their understanding of discharge instructions however, written provision of written information alone is not sufficient. An American study found a low proportion of older adults’ report adherence to medication instructions (35%) 4 days after ED discharge despite provision of written discharge instructions [54]. The reason for this may relate to our findings on the provision of appropriate information. We did identify examples of provision of written information on discharge in four included studies [28, 30, 31, 34]. But it was not clear if the information provided was a discharge summary written for other healthcare providers or written information designed for patients. However, criticisms of the written information such as it being too lengthy or difficulty to understand were also reported in all four of these studies. Given the small number of studies reporting provision of written information and the criticisms of same, healthcare staff should establish older adults’ health literacy and communication preferences to inform their use of written information. Furthermore, findings from this synthesis identified that older adults report communication challenges in their interactions with ED healthcare providers at discharge including use of medical jargon and provision of conflicting instructions/advice by healthcare providers, not having an opportunity to ask follow-up questions or seek clarity, and their sensory deficits not being accommodated for by healthcare providers. The European Task Force for Geriatric Emergency Medicine expert clinical recommendations include many recommendations that address concerns raised by older adults in this synthesis including the need to offer sensory aids and the need to ensure both the patient and carer understand arrangements related to discharge planning and any other follow up instructions [55]. Provision of an assistive listening device for older adults with hearing impairment in the ED is also recommended [55] and is associated with improved perception of quality of care in the ED [3]. A systematic review examining the effects of different manners of providing discharge information to ED patients concluded that verbal instructions alone may not be sufficient and adding video or written information to discharge instructions showed promising results [56]. Overall findings from this synthesis highlight that, healthcare providers need to optimise their communication style and methods when providing discharge instructions to older adults in the ED. We found that older adults wanted a planned discharge home with safe organised transport. The European Task Force for Geriatric Emergency Medicine recommended that clinicians ensure both the patient and carer understand arrangements related to discharge planning and any other follow up instructions [55]. In 2018, the American College of Emergency Physicians (ACEP) launched the Geriatric ED Accreditation (GEDA) program to accredit ED’s based on adherence to the Geriatric Emergency Department Guidelines [57]. Analysis of 225 ED’s in America that received accreditation revealed that, only 25 of these ED’s had access to transportation services for return home of older adults and 16 ED’s reported having a process for follow up after discharge [58]. Interventions such as Geriatric ED and comprehensive geriatric unit, focused care co-ordination with discharge planning and referral for community services are associated with improved older adult experience [3].

### Strengths and limitations

This study provided a rich and in-depth understanding of older adult’s experiences when transitioning from the ED to the community. The risk of neglecting potential articles was minimised by employing a broad search strategy and searching multiple databases. To support study rigour a protocol was pre-registered on PROSPERO. Search results were screened independently by two research members and strong inter-rater agreement was noted.

However, several limitations are associated with this study. Included studies in this synthesis were limited to English language as no transcription service was available. Secondly, studies included in the synthesis were all conducted in high income settings (Canada, Denmark, the United Kingdom, United States of America and the Netherlands). It could be suggested given these healthcare systems have significant funding and resources this may limit generalisability of the findings to other settings. Five of the included studies were conducted in Canada potentially further limiting generalisability. Limited contextual information is presented on models of transitional care, primary and community and other programs available to older adults during transition from the ED in the included studies to contextualise findings. Additionally, participants in the included studies may not represent the full diversity of older adults who attend the ED. Some included studies excluded non-English speaking older adults and older adults with sensory deficits or cognitive impairment. Of those studies that did not explicitly exclude older adults with cognitive impairment it was not clear how many participants with cognitive impairment participated. Thus, we do not know if the experiences of older adults with dementia are represented in the findings. We did not identify any study focused exclusively on the experiences of older adults’ transition from the ED to a nursing home. Data collection methods used in the included studies were a further limitation identified as none of the included studies used observation-based research methods which may be more appropriate to ensure the experiences of older adults with dementia are fully represented [59].

### Implications for practice/ research

This study highlighted that communication, care integration and symptom management are core issues that need to be addressed to improve the experience and care of older adults transitioning to the community from the ED. Further research is needed to explore the experiences of older adults with cognitive impairments at the point of transition from the ED to the community as none of the included studies focused on this population. Additionally, future research on transition from the ED to the community outside of high-income settings is needed. Further research on effective mechanisms of care integration and information sharing for older adults transitioning from the ED to the community is also needed.

## Conclusion

This study aimed to synthesis qualitative studies reporting older adults’ experiences of transition to the community from the ED. Ten studies representing the experiences of 334 older adult participants were included in the synthesis. Older adults commonly reported un-resolved symptoms on discharge which negatively affected transition to the community and return to daily life. Unresolved symptoms were also a major driver of early ED reattendance. Although older adults wanted a planned discharge home with organised transport this was infrequently achieved. Fragmented care was common as was inadequate healthcare provider communication. A key implication arising from this synthesis is the need for integrated healthcare systems and seamless transfer of health information to ensure safe transitions of care from the ED to the community. Healthcare providers should also adapt their communication and provide accessible information to meet the needs of older adults and explicitly address expectations about symptom resolution. Those developing transitional care interventions should consider the older adults need for integration of care and symptom management.

### Supplementary Information

**Additional file 1. **Search String/MESH Terms.

**Additional file 2. **eMERGe reporting guidelines.

**Additional file 3. **Characteristics and findings summary table.

**Additional file 4. **Discharge destination from ED of older adults reported in included studies.

**Additional file 5. **CASP Summary Table.

## Data Availability

The datasets used and/or analysed during the current study available from the corresponding author on reasonable request.

## Abbreviations

ED: Emergency Department
GP: General Practitioner
CASP: Critical Appraisal Skills Programme
PPI: Public and Patient Involvement
HIE: Health Information Exchange
