# Supplementary material for: Older adults experience of transition to the community from the emergency department: a qualitative evidence synthesis
Source: BMC Geriatr. 2024 Mar 6;24:233. doi: 10.1186/s12877-024-04751-6 (PMC10916040; doi:10.1186/s12877-024-04751-6)
Supplement: Supplementary file 1 — Additional file 1. Search String/MESH Terms. [file 12877_2024_4751_MOESM1_ESM.docx]

**Supplementary File 1 Search String/MESH Terms**

Search of Academic Search Complete, CINAHL, MEDLINE, PsycARTICLES, PsycINFO, and Social Science Full Text through EBSCOHost

AB ( Qualitative OR “case studies” OR “evaluation methods” OR interview* OR “focus group*” OR “Mixed methods” OR “naturalistic observation” OR “Participant observation” OR “social science research” OR “Consumer participation” OR transcript* OR ethnography* OR phenomenol* OR “grounded theory*” OR “purposive sample” OR hermeneutic* OR heuristic*OR semiotics OR “lived experience*” OR narrative*OR “life experience*” OR “life story*” OR “cluster sample” OR “action research” OR “observational method” OR “content analysis” OR “thematic analysis” OR “narrative analysis” OR “constant comparative method” OR “field stud*” OR “fieldnotes” OR “audio recording” OR “videorecording” OR “Theoretical sample” OR “discourse analysis” OR “Conversation analysis” ) AND AB ( “Aged People” OR “Aged Individual*” OR Aging OR Elderly OR Senior* OR Geriatric* OR “Older people” OR “Older Adult*” or “Older Individual*” OR “aged 65+” OR ‘65’ OR “aged sixty five” OR “over sixty-five” OR “Over 65” ) AND AB ( ‘Emergency department’ OR ‘emergency room’ OR ‘accident and emergency’ OR ‘accident & emergency’ OR ‘A & E’ OR ‘Casualty department’ OR ‘triage in the emergency department’ OR ‘triage’ OR ‘triage system’ )

**Limiters** - Publication Date: 20000101-20230331

**Expanders** - Apply equivalent subjects.

**Narrow by Language:**- English

**Search modes** - Boolean/Phrase

Search of Google Scholar: ‘Qualitative research’ AND ‘Emergency Department’ AND ‘Older Adults’.
