# Supplementary material for: Older adults experience of transition to the community from the emergency department: a qualitative evidence synthesis
Source: BMC Geriatr. 2024 Mar 6;24:233. doi: 10.1186/s12877-024-04751-6 (PMC10916040; doi:10.1186/s12877-024-04751-6)
Supplement: Supplementary file 2 — Additional file 2. eMERGe reporting guidelines. [file 12877_2024_4751_MOESM2_ESM.docx]

| No | Criteria Headings  **Supplementary File 2: eMERGe reporting guidelines.** | Reporting Criteria | Report Location |
| --- | --- | --- | --- |
| Phrase 1: Selecting the meta-ethnography and getting started | | | |
| Introduction | | | |
| 1 | Rational for context for meta-ethnography | Describe the gap in research or knowledge to be filled by the meta-ethnography, and the wider context of the meta-ethnography | Page 4 -6 |
| 2 | Aims of the meta-ethnography | Describe the meta-ethnography aim(s) | Page 6 |
| 3 | Focus of the meta-ethnography | Describe the meta-ethnography review question(s) (or objectives) | Page 6 |
| 4 | Rationale for using meta-ethnography | Explain why meta-ethnography was considered the most appropriate qualitative synthesis methodology | Page 9 |
| Phrase 2- Deciding what is relevant | | | |
| Methods | | | |
| 5 | Search strategy | Describe the rationale for the literature search strategy | Page 7 |
| 6 | Search Processes | Describe how the literature searching was carried out and by whom | Page 7 |
| 7 | Selecting primary studies | Describe the process of study screening and selection, and who was involved | Page 7 & 8 |
| Findings | | | |
| 8 | Outcome of the study | Describe the results of study searches and screening | Page 12 |
| Phrase 3- Reading Included studies | | | |
| Methods | | | |
| 9 | Reading and data extraction approach | Describe the reading and data extraction method and processes | Page 8 & 9 |
| Findings | | | |
| 10 | Presenting characteristics of included studies | Describe the characteristics of the included studies | Supplementary file 3 & page 13 |
| Phrase 4- Determining how the studies are related | | | |
| Methods | | | |
| 11 | Process for determining how studies are related | Describe the methods and processes for determining how the included studies are related:  Which aspects of studies were compared  AND  How the studies were compared | Page 9 & 10 |
| Findings | | | |
| 12 | Outcome of relating studies | Describe how the studies relate to each other | Page 14-24  Supplementary file 3 |
| Phrase 5-Translating studies into one another | | | |
| 13 | Process of translating studies | Describe the methods of translation:  -Describe steps taken to preserve the context and meaning of the relationships between concepts within and across studies- Describe how the reciprocal and refutational translations were conducted- Describe how potential alternative interpretations or explanations were considered in the translations | Page 9 & 10 |
| Findings | | | |
| 14 | Outcome of translation | Describe the interpretive findings of the translation. | Page 14-24 |
| Phrase 6- Synthesizing translations | | | |
| Methods | | | |
| 15 | Synthesis Process | Describe the methods used to develop overarching concepts (“synthesised translations”)  Describe how potential alternative interpretations or explanations were considered in the synthesis | Page 9 & 10 |
| Findings | | | |
| 16 | Outcome of synthesis process | Describe the new theory, conceptual framework, model, configuration or interpretation of data developed from the synthesis | Page 14-24  Page 10 |
| Phrase 7-Expressing the synthesis | | | |
| Discussion | | | |
| 17 | Summary of findings | Summarize the main interpretive findings of the translation and synthesis and compare them to existing literature | Page 26-30 |
| 18 | Strengths, limitations, and reflexivity | Reflect on and describe the strengths and limitations of the synthesis:  - Methodological aspects—for example, describe how the synthesis findings were influenced by the nature of the included studies and how the meta-ethnography was conducted.  - Reflexivity—for example, the impact of the research team on the synthesis findings | Page 30 & 31  Page 6 & 7 |
| 19 | Recommendations and conclusion | Describe the implications of the synthesis | Page 31 |
