# Supplementary material for: Older adults experience of transition to the community from the emergency department: a qualitative evidence synthesis
Source: BMC Geriatr. 2024 Mar 6;24:233. doi: 10.1186/s12877-024-04751-6 (PMC10916040; doi:10.1186/s12877-024-04751-6)
Supplement: Supplementary file 3 — Additional file 3. Characteristics and findings summary table. [file 12877_2024_4751_MOESM3_ESM.docx]

| **Citation**  **Supplementary File 3 Characteristics and findings summary table** | **Country/Setting** | **Year** | **Population** | **Sample** | **Aim** | **Methodology/Data Collection Method** | **Data Analysis** | **Themes** |
| --- | --- | --- | --- | --- | --- | --- | --- | --- |
| Cetin-Sahin et al 2020 | Canada  4 University-affiliated EDs between Montreal and Quebec City | 2020 | Older Adults aged 75 and older discharged to their original residence (own home, residence, nursing home) from ED. An accompanying proxy was invited to participate if a patient was too ill, cognitively impaired, or not able to communicate in English or French. | Purposeful Subsample used for Qualitative Analysis comprised:  108 older adults (71.3% female)  32 proxies  (26 children, 5 spouses and 1 friend). | To explore experiences of an ED visit among patients aged 75 and older. | Mixed Methods Study including both data collected via structured telephone interviews and qualitative data.  Qualitative data collected via telephone interviews with open ended questions 1 week after discharge. | A hybrid deductive-inductive approach to thematic analysis was conducted.  Only data analysed deductively reported in this paper. | Findings categorised under 3 domains:  (1) Older Patients’ Physical needs  (2) The needs of family members  (3) Transitional Care Needs |
| Dresden et al 2019 | United States of America  Urban Academic ED | 2019 | Older Adults >65 years or older who had been discharged from the ED within 45 days prior to start of recruitment. | N= 31  Women: 70%  Men: 30%  Median age: 70 (interquartile range = 67–74) | To understand which aspects of Health-Related Quality of life (HRQoL) are most valued by geriatric patients in the ED and what expectations patients have for addressing or improving HRQoL during an ED visit. | Data collected via 6 focus groups (60 min duration) (between March 2013 and June 2013).  Data from three pilot semi-structured interviews conducted to develop the focus group script were also analysed. | Latent content and constant comparative methods. | Five themes identified: (1) Functional Recovery (2) Mental health and anxiety (3) Interpersonal effects of health on quality of life (4) Individual experience of health and quality of life (5) Is the ED the right place to discuss quality of life. |
| Gettel et al 2022 | USA  Four hospital EDs: a Level I trauma centre/tertiary referral hospital, two academic community hospitals, and a freestanding ED within the same health system. | 2022 | Patients that (1) aged 65 years or older, (2) anticipated discharge after the ED encounter, and (3) fluent in English or Spanish | N=25  20% male participants  Age, mean years: 72.2 | To assess barriers experienced by older adults during ED-to-community care transitions | Participants were contacted within 3–7 days of the initial ED visit. One to one interview using a semi-structured interview guide (based on existing literature, study teams’ expertise and a newly developed conceptual framework of ED to community care transitions). | Iterative process of thematic analysis (including both inductive and deductive coding) to synthesize the data, identify patterns, and develop themes across the interviews.  Researchers constructed a conceptual framework a priori to guide coding. | Identified four barriers during the ED-to-community care transition: (1) ED discharge process was abrupt with missing information (2) navigating follow-up outpatient clinical care was challenging, (3) new physical limitations and fears hinder function (4)  acceptance of formal and informal assistance. |
| Goodridge et al 2018 | Canada,  Participants were recruited using announcements and postings from agencies whose users were compromised of primarily older adults. | 2018 | Older Adults’ >65 and had attended an ED within an urban centre within the preceding 2 years. Caregivers who had accompanied an older adult to the ED within the past 2 years. | ED patients N=41  Female: 92.9%  Caregivers N=15  Female: 93% | Describe the patient experience of older adults in the ED and generate recommendations for enhancing their experience. | Qualitative Descriptive Design.  Ten Focus Groups (June 2017).  Two participants, who attended an urban ED but resided outside of commuting distance participated in individual interviews. | Text driven approach to thematic analysis. | (1) Health system and provider factors affecting the ED experience of older adults, (2) Older adults’ strategies for negotiating the patient experience in the ED, (3) Key recommendations for enhancing the older adult experience in the ED. |
| Kolk et al 2021 | Netherlands  Level 1 Trauma Centre ED | 2021 | Older Adults >70 who frequently visited the ED and were discharged home after their last visit. Medical history of two or more morbidities and a previous visit to the ED or hospital during the past 18 months. | N=13 (Thirty-two older adults were screened for eligibility)  Male: N= 6  Female: N= 7  Mean age (SD)= 75(6)  Range: 70-91 | To describe older patients’ perspectives and experiences before and after and ED visit, and to identify factors that possibly contribute to frequent ED visits. | Qualitative Description study.  Semi-Structured Interviews lasting approx. 90 minutes in participants home between 7 and 30 days after discharge.  Data collected between June and Sept 2019. | Theoretical Analysis (form of thematic analysis) | Three Main themes identified: (1) Medical events leading to feeling of crisis (2) Patients’ untreated health problems (3) Persistent problems in health and daily functioning post discharge. |
| Marr et al  2019 | Canada  ED at a large urban Canadian hospital. | 2019 | Older Adults > 65 able to read, write, and speak in English; cognitively intact as determined by medical staff; living independently in the community (own home, retirement home) and were to be discharged home from ED.  Caregivers had to primary caregiver of someone who was admitted to the ED; they also had to be present in the ED and read, write, and speak English. | Mean age of ED patients: 81.1years  N=51 older adults  60.8% female  N=25 Caregivers (6 partners / spouses 19 children). | To learn more about the perspectives of older adults and their caregivers on their experiences transitioning to the community following an ED visit. | Two-stage Methodology  Survey in the ED (quantitative)- findings not included in this synthesis.  Structured interviews (Memory Impairment screen was conducted). Interview included responses to closed items with rating scale and open-ended questions. | Naturalistic inquiry approach | Six categories were generated: (1) Communication (2) Limited understanding of health condition (3) Availability of caregiver support (4) Patient resistance to accept recommendations (5) Inadequate support for caregivers (6) Various external factors. |
| Nielsen et al 2019 | Denmark  University Hospital ED | 2019 | Older Adults >65 who had experienced an intervention designed to improve discharge practices in an experimental study who were acutely admitted and discharged directly to their own home from a short-stay unit at the ED and living in a larger municipality in Denmark (qualitative study took place alongside a quasi-experimental study). | N=11  Male: N=3  Female: N=8 | To explore and gain a deeper understanding of elderly patients’ experiences of being discharged and their return to everyday lives after discharge from a short-stay unit the ED. | Descriptive Phenomenological Methodology.  Semi-structured interview lasting 30-60min two weeks after participants were discharged. | Malterud’s modification of Giorgi’s phenomenological approach to systematic text condensation. | Four themes emerged: (1) Pain and fatigue limited performance of daily activities (2) Frustrations and concerns (3) The importance of being involved and listened to during admission (4) The importance of being prepared for being discharged. |
| Phelps et al 2022 | United Kingdom  Three ED clinical sites | 2022 | Participants were included if they had mild or greater frailty (≥5 on the Clinical Frailty Scale). Informed consent was taken by staff who recruited participants within 72 hours of participants’ entry to ED, with the majority being recruited on the day of attendance. | Caregivers N=26  Older adults: N=24  Female: 84.2%  (Caregivers and older adults reported on episodes of urgent care which started in ED for 28 patients). | To examine the views of older people living with frailty and their families in relation to specific episodes of urgent care, what they wanted to achieve and whether those goals were attained. | Semi-structured in-depth interview conducted by two experienced non-clinical qualitative researchers (60 minutes duration).  Interviews in the majority of cases were conducted within 30 days of the ED attendance. | The Framework Approach | Findings are presented as goals of care and attainment of those goals:  (1) Physical and medical goals of care (2) Function and Mobility (3) Ongoing treatment and care (4) Discharge and discharge planning (5) Goals of care concerning information and involvement in decision making |
| Uscatescu et al 2014 | Canada  ED at a large Canadian urban hospital | 2014 | Older Adults > 65 with any diagnosis who returned for an unscheduled visit to the ED within 14 days of a previous ED visit. | N=15  Women: N=7  Men: N=8  Mean Age: 80 years | To explore the factors that led to early return visits to the ED by older adults. | Qualitative descriptive design  Semi-structured interviews. Interview conducted by Geriatric Liaison Nurse (between September and December 2011) | Colaizzi’s seven steps of analysis | Three Themes were identified: (1) Managing the symptoms (2) Care during the Initial ED visit (3) Who I am. |
