# Supplementary material for: Older adults experience of transition to the community from the emergency department: a qualitative evidence synthesis
Source: BMC Geriatr. 2024 Mar 6;24:233. doi: 10.1186/s12877-024-04751-6 (PMC10916040; doi:10.1186/s12877-024-04751-6)
Supplement: Supplementary file 4 — Additional file 4. Discharge destination from ED of older adults reported in included studies. [file 12877_2024_4751_MOESM4_ESM.docx]

Supplementary File 4: Discharge destination from ED of older adults reported in included studies

| **Study** | **Sample Size** | **Own home or other family members home** | **Retirement home/Care Home** | **Senior Living facility** | **Assisted Living /** | **Other** |
| --- | --- | --- | --- | --- | --- | --- |
| Boye et al (2021) | N=15 | N=15  (Lived alone N=11 Lived with spouse N=4­) |  |  |  |  |
| Cetin-Sahin et al (2020) | N=108 | N=65 |  |  |  | N=43 (described as senior residence, nursing home or rehabilitation center) |
| Dresden et al (2019) | N=31 No breakdown of participants living situation provided. | | | | | |
| Gettel et al (2022) | N=25 No breakdown of participants living situation provided, study reported participants were community dwelling. | | | | | |
| Goodridge et al (2018) | N=41 No breakdown of participants living situation provided. | | | | | |
| Kolk et al (2021) | N=13 No breakdown of participants living situation provided, study reported participants were discharged home. | | | | | |
| Marr et al (2019) | N=51 | N=45  (Lived alone N=27  Lived with others N=24) | N=3  (Retirement home) |  | N=3 |  |
| Nielson et al (2019) | N=11 | N=11  (Lived alone N=8  Living with partner N=3) |  |  |  |  |
| Phelps et al (2022) |  | N=24  (Lived alone or with spouse N=18  Lived with other family members N=4  Lived with live in carers N=1) | N=2  (Care home) |  | N=3  Sheltered accommodation |  |
| Uscatescu et al (2014) | N=15 | N=13  (Lived alone N=2  Lived with family N=11) |  | N=2 |  |  |
