# Supplementary material for: Older adults experience of transition to the community from the emergency department: a qualitative evidence synthesis
Source: BMC Geriatr. 2024 Mar 6;24:233. doi: 10.1186/s12877-024-04751-6 (PMC10916040; doi:10.1186/s12877-024-04751-6)
Supplement: Supplementary file 5 — Additional file 5. CASP Summary Table. [file 12877_2024_4751_MOESM5_ESM.docx]

| Authors | Question1  Was there a clear statement of the aims of the research? | Question 2  Is a qualitative methodology appropriate? | Question 3  Was the research design appropriate to address the aims of the research? | Question 4  Was the recruitment strategy appropriate to the aims of the research? | Question 5  Was the data collected in a way that addressed the research issue? | Question 6  Has the relationship between researcher and participants been adequately considered? | Question 7  Have ethical issues been taken into consideration? | Question 8  Was the data analysis sufficiently rigorous? | Question 9  Is there a clear statement of findings? |
| --- | --- | --- | --- | --- | --- | --- | --- | --- | --- |
| Boye et al 2021 | Yes | Yes | Yes | Yes | Can’t tell | No | Yes | Yes | Yes |
| Cetin Sahin et al 2020 | Yes | Yes | Yes | Yes | Can’t tell | No | Yes | Can’t tell | Yes |
| Dresden et al 2019 | Yes | Yes | Can’t tell | Yes | Can’t tell | Can’t tell | Yes | Yes | Yes |
| Gettel et al 2022 | Yes | Yes | Yes | Yes | Yes | Yes | Yes | Yes | Yes |
| Goodridge et al 2018 | Yes | Yes | Can’t tell | Yes | Can’t tell | Yes | Yes | No | Yes |
| Kolk et al 2021 | Yes | Yes | Yes | Yes | Yes | Yes | Yes | Yes | Yes |
| Marr et al 2019 | Yes | Yes | Can’t tell | Yes | Can’t tell | Can’t tell | Yes | Yes | Yes |
| Nielsen et al 2019 | Yes | Yes | Yes | Can’t tell | Yes | Yes | Yes | Yes | Yes |
| Phelps et al 2022 | Yes | Yes | Yes | Yes | Yes | Yes | Yes | Yes | Yes |
| Uscateau et al 2014 | Yes | Yes | Yes | Yes | No | No | Can’t tell | Can’t tell | Yes |

**Supplementary File 5: CASP Summary Table**
